# Reexperiencing and anxious arousal symptoms in relation to volumes of thalamus nuclei in posttraumatic stress spectrum adults

**DOI:** 10.1002/brb3.2639

**Published:** 2022-06-08

**Authors:** Emily J. Casteen, Sienna R. Nielsen, Elizabeth A. Olson, Kevin Frederiks, Isabelle M. Rosso

**Affiliations:** ^1^ Center for Depression Anxiety and Stress Research McLean Hospital Belmont MA USA; ^2^ Department of Psychiatry Harvard Medical School Boston MA USA

**Keywords:** adult, arousal, episodic, memory, posttraumatic, stress disorders, thalamic nuclei, thalamus

## Abstract

**Introduction:**

Trauma reexperiencing is dominated by recollection of sensory‐perceptual elements of the trauma, pointing to involvement of the sensory thalamus. This study examined posttraumatic stress symptoms in relation to volumes of thalamic nuclei that were grouped based on their predominant functions. We hypothesized that reexperiencing, controlling for other symptom dimensions, would correlate with volumes of thalamic nuclei involved in primary and higher‐order sensory processing.

**Methods:**

Seventy‐two trauma‐exposed adults were interviewed with the Clinician Administered PTSD Scale for DSM‐IV and underwent 3T magnetic resonance imaging. Scores were derived for reexperiencing, anxious arousal, dysphoric arousal, emotional numbing, and avoidance symptoms. These were entered as simultaneous predictors in five separate regression analyses, with age, sex, and total thalamus volume as covariates, predicting volumesf of five thalamus nuclear groupings corrected for intracranial volume: Specific sensory, associative‐sensory, associative‐cognitive, intralaminar, and motor groupings.

**Results:**

Reexperiencing symptoms were significantly positively correlated with volumes of the motor thalamic grouping, which included the ventral anterior, ventral lateral, and ventromedial nuclei. Anxious arousal was significantly negatively correlated with volumes of all five thalamic groupings.

**Conclusions:**

Reexperiencing symptoms were correlated with volumes of the motor thalamus, while anxious arousal symptoms were related to all thalamic subregion volumes. Thalamic nuclei involved in motor functions, including oculomotor control and motor planning, may be implicated in posttraumatic reexperiencing symptoms.

## Graphical abstract

Posttraumatic stress disorder involves reexperiencing of a traumatic experience, including its sensory‐perceptual elements. This study examined reexperiencing and other posttraumatic stress symptoms in relation to volumes of thalamic nuclei that were grouped based on their role in particular functions (sensory, cognitive, motor, arousal). We found that reexperiencing symptoms were associated with volumes of the motor thalamus, while anxious arousal symptoms are related to the size of all thalamic subregion volumes. These results suggest that thalamic nuclei involved in oculomotor control and motor planning may be implicated in posttraumatic reexperiencing symptoms.

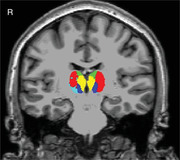

## 1 INTRODUCTION

Intrusive reexperiencing symptoms are core to posttraumatic stress disorder (PTSD) and are often dominated by recollection of sensory‐perceptual elements of the trauma (Brewin, 2014). This phenomenology points to involvement of sensory brain regions such as the thalamus, a key hub for sensory information processing in the brain. In neurocircuitry models, trauma reexperiencing is proposed to arise from an implicit fear‐learning process in which sensory information about the threat has been passed directly from the thalamus to the amygdala, bypassing cortical regions that would have assisted in consciously processing the information (Bremner et al., 1995; Lanius et al., 2017; LeDoux, 2000; Liberzon et al., 1999; Neumeister et al., 2007; Rauch et al., 2006). The threat‐related information that is processed through this subcortical thalamus–amygdala pathway (Lanius et al., 2017) is subsequently stored and recalled involuntarily as fragmented sensory information, rather than as an integrated autobiographical memory of the event (Brewin, 2014). Some theories (Lanius et al., 2017; Neumeister et al., 2007) emphasize the role of thalamic nuclei that are specifically involved in sensory information processing, such as the pulvinar nucleus (Lanius et al., 2017).

The thalamus is a heterogeneous structure consisting of distinct nuclei with different anatomical connectivity and functions (Bocchetta et al., 2020; Greene et al., 2020; Haber, 2003; Halassa & Kastner, 2017; Hallock et al., 2016; Herrero et al., 2002; Hwang et al., 2017; Iglesias et al., 2018; Ilyas et al., 2019; Purpura & Schiff, 1997; Van der Werf et al., 2002). Some thalamic nuclei serve as primary sensory relays (“specific sensory nuclei,” Table 1), including the lateral geniculate nucleus, medial geniculate nucleus, and ventral posterolateral nucleus. These relay nuclei receive peripheral sensory information and transfer it to primary visual, auditory, and somatosensory cortices. A second functional grouping of ventral thalamic nuclei can be distinguished based on its principal involvement in motor functions (“motor nuclei”), including the ventral anterior, ventral lateral, and ventromedial nuclei. Third, the intralaminar grouping of thalamic nuclei (“intralaminar nuclei”) is involved in general arousal, alerting, and pain processing. Finally, there are thalamic nuclei that support higher‐order associative and limbic functions, including some involved in higher‐order sensory processing (pulvinar, lateral posterior: “associative‐sensory nuclei”) and others in learning, memory, and aspects of executive functioning (anteroventral, laterodorsal, mediodorsal, reuniens, and paratenial: “associative‐cognitive nuclei”). These nuclear groupings can be derived from the magnetic resonance imaging (MRI)‐based automated parcellation of the thalamus using a recent version of Freesurfer software (Iglesias et al., 2018), as has been done in a study of frontotemporal dementia (Bocchetta et al., 2020). To our knowledge, functional groupings of thalamic nuclei have not yet been examined in relation to PTSD symptoms.

**TABLE 1:** Groupings of thalamic nuclei along with their predominant functions (Bocchetta et al., 2020; Halassa and Kastner, 2017; Hallock et al., 2016; Hwang et al., 2017; Purpura and Schiff, 1997; Ilyas et al., 2019; van der Werf et al., 2002), and the individual nuclei in each grouping (Iglesias et al., 2018)

Grouping	Functions	Individual Nuclei
Specific sensory	Primary visual, auditory, and somatosensory	LGN, MGN, VPL
Associative‐sensory	Higher‐order visual, somatosensory, visual–spatial integration	Pul, LP
Associative‐cognitive	Learning, memory, cognition, emotion regulation	AV, LD, MD, MV‐re, Pt
Intralaminar	Arousal, alerting, pain	CeM, CL, Pc, CM, Pf
Motor	Motor, oculomotion, motor planning	VA, VL, VM

Abbreviations: AV, anteroventral; CeM, central medial; CL, central lateral; CM, centromedian; LD, laterodorsal; LGN, lateral geniculate nucleus; LP, lateral posterior; MGN, medial geniculate nucleus; MD, mediodorsal; MV‐re, reuniens (medial ventral); Pc, paracentral; Pf, parafascicular; Pul, pulvinar; Pt, parateniens; VA, ventral anterior; VL, ventral lateral; VM, ventromedial.

Most previous anatomical studies of the thalamus in PTSD have considered the thalamus in its entirety, rather than individual thalamic nuclei or groupings of functionally homogeneous nuclei. There have been varied reports of smaller whole thalamus volume in individuals with PTSD compared to controls (Cardenas et al., 2011; Nardo et al., 2010; O'Doherty et al., 2017), or no significant group difference (Chen et al., 2012; Landré et al., 2010; Mutluer et al., 2018; Sussman et al., 2016). A meta‐analysis from the ENIGMA consortium showed that volumes of the whole thalamus do not differ significantly between PTSD patients and trauma‐exposed control subjects (Logue et al., 2018). The mixed nature of these anatomical findings could derive from two limitations of the previous research: (a) Examination of the whole thalamus rather than thalamic nuclei and (b) few reports on the relation between thalamus volumes and PTSD symptom dimensions.

To our knowledge, only one anatomical study has examined individual thalamic nuclei in a PTSD sample. Chen et al. (2012) reported on the pulvinar, which is the largest thalamic nucleus. They found that survivors of a coal mining disaster who had not developed PTSD within 3 months of the event (*n* = 10) had a smaller right pulvinar compared with nontraumatized controls (*n* = 10). In contrast, the coal miners who had developed PTSD following the disaster (*n* = 10) did not differ from nontraumatized controls (*n* = 10) on pulvinar volume. Although these results could suggest that a smaller pulvinar nucleus of the thalamus is associated with less risk of developing PTSD symptoms following trauma exposure, the study's small sample precludes drawing strong conclusions. Another limitation of most prior studies of thalamus volume in PTSD is that they considered PTSD as a homogeneous illness, without investigating potential differential relationships between thalamus volume and PTSD symptom dimensions. In a study that did examine PTSD symptom dimensions in relation to thalamus anatomy, Shucard et al. (2012) reported that reexperiencing was the only symptom cluster significantly associated with (whole) thalamus volume, showing a significant negative correlation with left thalamus volume. Additional research is therefore needed to characterize the relationship between PTSD symptoms and anatomical variation in thalamic nuclei.

In the current study, we examined trauma‐exposed adults along a dimensional spectrum of PTSD symptom severity, ranging from mild and subthreshold symptoms through severe symptoms that met criteria for the clinical diagnosis. The premise of this approach is that reliance on categorical diagnostic phenotypes is not as well suited to identifying pathophysiologic correlates as a dimensional model that captures the full range of syndromal variation (McLean et al., 2020). Indeed, selecting only participants who meet for the clinical PTSD diagnosis, and excluding subthreshold individuals, yields a truncated range of symptom variation and reduced generalizability of findings. Accordingly, we did not restrict our examination of reexperiencing symptoms correlates to people who currently met the clinical threshold for DSM‐based PTSD. Instead, consistent with a dimensional Research Domain Criteria approach, we included participants with a full range of variation of posttraumatic stress symptomatology (Cuthbert & Insel, 2013).

Given involvement of sensory processes in intrusive reexperiencing, we hypothesized that reexperiencing symptoms would be associated with volumes of thalamic nuclei involved in sensory processing within trauma‐exposed posttraumatic stress adults. Based on thalamus divisions derived by Bocchetta et al. (2020) (Table 1), we combined individual thalamic nuclei into five major groupings related to their functional specializations: Specific sensory, associative‐sensory, associative‐cognitive, intralaminar, and motor. We hypothesized that reexperiencing symptoms, controlling for other PTSD symptom dimensions, would be significantly associated with volumes of the specific sensory nuclei involved in primary sensory functions and with volumes of the associative‐sensory nuclei involved in higher‐order sensory processing. In addition, based on evidence that a five‐factor dysphoric arousal model of PTSD symptoms may better represent the symptom structure and neurobiological underpinnings of PTSD than three‐ and four‐factor models (Pietrzak et al., 2012; Pietrzak, Galea, et al., 2013; Pietrzak, Henry, et al., 2013), we grouped PTSD symptoms into five subscales: Reexperiencing, emotional numbing, avoidance, dysphoric arousal, and anxious arousal symptoms.

## 2 METHODS

### 2.1 Participants

Seventy‐two trauma‐exposed adults (38 women) were recruited from the greater Boston metropolitan area. All participants met criteria for at least one PTSD Criterion A trauma exposure, and had varying symptoms of posttraumatic stress, thereby falling on a continuum of PTSD symptom severity. Twenty‐three (23) met criteria for current PTSD, 15 for lifetime PTSD, and 34 had never met full criteria for PTSD. All subjects received a full explanation of study procedures and provided written informed consent. Inclusion criteria were: (1) Right‐handedness; (2) aged 20–50 years; and (3) exposure to a DSM‐IV PTSD Criterion A trauma. Exclusion criteria were: (1) Medical condition that would confound results; (2) history of seizures or serious head trauma with loss of consciousness; (3) metal implants, claustrophobia, or other MRI exclusions; (4) color blindness; (5) positive urine toxicology or pregnancy test; (6) history of psychotic disorder, bipolar disorder, eating disorder, mental retardation, pervasive developmental disorder, or obsessive‐compulsive disorder; and (7) current panic disorder. A stable dose of antidepressant medication was allowable, and one participant reported taking fluoxetine (50 mg per day). No other participants were taking psychotropic medications. The study was approved by the Partners Healthcare Human Research Committee.

### 2.2 Interviews and measures

#### 2.2.1 Clinical interviews

Participants underwent two semistructured clinical interviews, administered by doctoral‐level psychologists. The Structured Clinical Interview for DSM‐IV Axis‐I Disorders (SCID) assessed current and lifetime histories of other psychiatric disorders (First et al., 2002).

The Clinician‐Administered PTSD Scale, Current and Lifetime Versions (CAPS), yielded diagnoses of DSM‐IV PTSD and scores for PTSD symptom subscales (Blake et al., 1995). Index traumas were reported as violent assaults (*n* = 19, 26%), sexual assaults (*n* = 12, 17%), childhood maltreatment (*n* = 12, 17%), motor vehicle accidents (*n* = 10, 14%), mass violence (*n* = 6, 8%), witnessing violent death (*n* = 5, 7%), domestic violence (*n* = 3, 4%), combat (*n* = 2, 3%), natural disasters (*n* = 2, 3%), torture (*n* = 1, 1%). Scores for the five CAPS symptom subscales were defined as follows: (1) Reexperiencing (sum of all items from Criterion B), avoidance (sum of C1 and C2 from Criterion C), emotional numbing (sum of C3, C4, C5, C6, and C7 from Criterion C), dysphoric arousal (sum of D1, D2, and D3 from Criterion D), and anxious arousal symptoms (sum of D4 and D5 of Criterion D; Pietrzak et al., 2012).

#### 2.2.2 Self‐report questionnaires

The Life Events Checklist (LEC; (Gray et al., 2004)) is a 17‐item self‐report questionnaire of potentially traumatic events. LEC score was used as a measure of lifetime trauma exposure (number of personally experienced and personally witnessed Criterion A events).

The Childhood Trauma Questionnaire (CTQ; (Bernstein & Fink, 1998)) is a 28‐item self‐report questionnaire of childhood trauma exposures, including emotional neglect and abuse, physical neglect and abuse, and sexual abuse. We derived a total CTQ score as the sum of all reported childhood traumas.

The Beck Depression Inventory, version 1a (BDI; Beck & Steer, 1993) is a 21‐item self‐report inventory of depression symptoms experienced over the past 2 weeks. Total BDI score was used as a measure of depression severity.

### 2.3 MRI Imaging

#### 2.3.1 MRI data acquisition

Scans were acquired on a 3‐Tesla Siemens Tim Trio Scanner (Siemens, Erlangen, Germany), using a 32‐channel phased‐array design RF head coil operating at 123 MHz. Anatomical whole brain images were obtained, including T1‐weighted MPRAGE using parameters that were optimized for morphometric analysis on Freesurfer software (Dale et al., 1999; Fischl et al., 2002, 2004): 128 slices, echo time = 3.31 ms, repetition time = 2530 ms, inversion time = 1100 ms, flip angle = 7°, slice thickness = 1.33 mm (voxel size: 1.0×1.0×1.33 mm).

#### 2.3.2 MRI data processing

Images were processed using the “recon‐all” function of Freesurfer 6.0.1 software. This function runs multiple imaging processing steps including skull‐stripping, segmentation of subcortical brain areas, and parcellation of cortical regions. The recon‐all output was checked manually (EAO) for errors following the automated routine; this included verifying that subcortical structures including the whole thalamus were captured within the segmentation outline and that voxels outside the thalamus were not (as in Huang et al., 2020). The recon‐all output yielded measures of estimated intracranial volume (ICV), and whole thalamus volume. After manual quality control of the recon‐all output, we ran the automated subfield segmentation pipeline that derives volumes of 14 thalamic nuclei using the development version of Freesurfer (July 09, 2019 build). These thalamic nuclei were previously defined based on coregistrations of histological data and MRI images, have shown very high test‐retest reliability, and have been validated against manual delineations of ex‐vivo MRI scans of autopsy samples (Iglesias et al., 2018).

#### 2.3.3 Thalamus nuclei groupings

**FIGURE 1:**
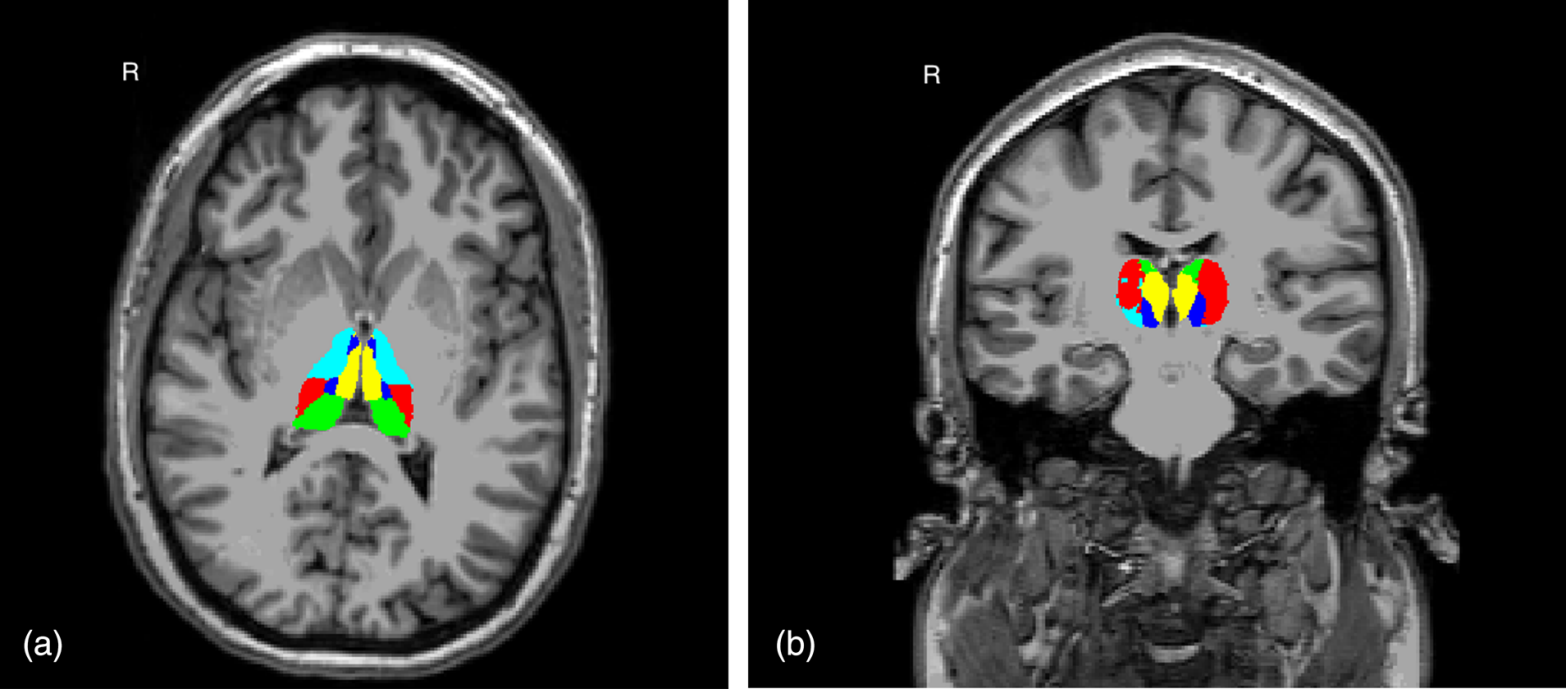
Thalamus nuclei groupings shown in axial (1a) and coronal (1b) views of an individual participant's T1 native space: Specific sensory (red), associative‐sensory (green), associative‐cognitive (yellow), intralaminar (dark blue), and motor (teal). R: right

Based on Bocchetta et al. (2020), we summed volumes of individual thalamic nuclei into functional groupings (Table 1, Figure 1). All volumetric measures were expressed as a ratio of total ICV to normalize for head size: (absolute structural volume / ICV).

### 2.4 Statistical analyses

In the absence of hypotheses about laterality, left and right hemisphere volumes of each thalamus grouping (specific sensory, associative‐sensory, associative‐cognitive, intralaminar, motor) were summed (Barnes et al., 2010; Whitwell et al., 2001). Five regression analyses used as dependent variables the volume of each thalamus grouping adjusted for ICV, applying a Bonferroni‐corrected alpha of .01. The five CAPS‐based symptom subscales for current symptom scores were entered as simultaneous independent variables in each regression. Effects of age and sex were used as covariates because these factors may confound between‐subject comparisons in volumetric brain imaging studies generally (Barnes et al., 2010), and have been associated with variation in thalamus volume specifically (Li et al., 2014; Wang et al., 2019). Total thalamus volume (“thalamus proper”) was entered as a covariate in the analyses following the same rationale and precedent set by Morey et al. (2020) in their examination of amygdala nuclei volumes in PTSD. We assessed for multicollinearity among symptom predictors using variation inflation factors (VIFs). We considered a VIF > 5 indicative of problematic collinearity (Kim, 2019) as more conservative than a commonly used VIF threshold of 10 (Myers, 1990). All model residuals were tested for normality. For significant findings, we examined potentially confounding effects of depression (BDI score) and trauma load (LEC score, CTQ score) in follow‐up regressions. Statistical analyses were performed using JMP 14 software (SAS Institute Inc).

## 3 RESULTS

### 3.1 Sample characteristics

Table 2 displays demographic and clinical characteristics of the sample.

**TABLE 2:** Demographic and clinical characteristics of the sample of trauma‐exposed adults (*N*, % or Mean ± Standard Deviation)

Characteristic	*N*, % or Mean ± SD
*N* (Female)	72 (38, 52.78%)
Age	33.69 ± 8.22
Education	
High school	17, 23.61%
College	36, 50.00%
Graduate school	19, 26.39%
Race	
Asian	8, 11.11%
Black	14, 19.44%
Other	4, 5.56%
White	46, 63.89%
Hispanic	8, 11.11%
CAPS Total Current	26.07 ± 25.13
CAPS Anxious Arousal	3.83 ± 4.04
CAPS Avoidance	3.64 ± 4.22
CAPS Dysphoric Arousal	4.64 ± 5.64
CAPS Emotional Numbing	7.71 ± 9.37
CAPS Reexperiencing	6.24 ± 7.03
BDI	9.65 ± 9.70
LEC	7.55 ± 4.1
CTQ	60.01 ± 23.04
Non‐PTSD Axis I Disorders	
Any depressive disorder	16, 23.61%
Major depressive disorder	12, 16.67%
Depressive disorder NOS	2, 2.99%
Dysthymic disorder	3, 4.48%
Any Anxiety disorder	12, 16.67%
Anxiety disorder NOS	1, 1.49%
Generalized anxiety disorder	3, 4.48%
Social phobia	2, 2.99%
Specific phobia	6, 8.33%

Abbreviations: CAPS: Clinician Administered PTSD Scale; BDI: Beck Depression Inventory, 1a; CTQ: Childhood Trauma Questionnaire; LEC: Life Events Checklist; PTSD: posttraumatic stress disorder.

### 3.2 PTSD symptom correlates of thalamus nuclei volumes

Table 3 shows results of the five regressions examining PTSD symptoms as predictors of thalamus grouping volumes. Reexperiencing symptom scores were significantly positively correlated with volumes of the motor thalamic nuclei. Anxious arousal symptoms were significantly negatively correlated with volumes of all thalamic groupings (specific sensory, associative‐sensory, associative‐cognitive, intralaminar, motor). In addition, female participants had significantly larger specific sensory, associative‐sensory, and motor thalamic volumes than male participants. Total thalamus volume was not a significant covariate in any of the five regressions. VIFs for the symptom scales ranged from 1.78 to 3.52, which are lower than thresholds of 5 to 10 taken as moderate to severe collinearity (Kim, 2019).

**TABLE 3:** Regression analyses examining posttraumatic stress symptom dimensions as predictors of volumes of thalamic nuclei

Thalamus Grouping	Predictor	Standardized β	*T*	*p*
Specific sensory	Anxious arousal	–0.554	–4.02	**.0002**
	Avoidance	0.189	1.20	.236
	Dysphoric arousal	0.055	0.28	.779
	Emotional numbing	0.083	0.51	.615
	Reexperiencing	0.381	2.36	.021
	Age	0.156	1.32	.193
	Sex[F]	0.364	3.07	**.003**
	Total thalamus	0.219	1.79	.079
Associative‐sensory	Anxious arousal	–0.451	–3.25	**.002**
	Avoidance	0.141	0.89	.376
	Dysphoric arousal	0.207	–1.06	.293
	Emotional numbing	0.271	1.64	.106
	Reexperiencing	0.359	2.22	.030
	Age	0.161	1.35	.183
	Sex[F]	0.357	3.00	**.004**
	Total thalamus	0.007	0.05	.957
Associative‐cognitive	Anxious arousal	–0.481	–3.31	**.002**
	Avoidance	0.122	0.74	465
	Dysphoric arousal	–0.116	–0.57	.572
	Emotional numbing	0.222	1.28	.204
	Reexperiencing	0.398	2.35	.022
	Age	0.065	0.52	.604
	Sex[F]	0.304	2.44	.018
	Total thalamus	0.150	1.16	.249
Intralaminar	Anxious arousal	–0.502	–3.62	**.0006**
	Avoidance	0.186	1.17	.2466
	Dysphoric arousal	–0.147	–0.75	.454
	Emotional numbing	0.247	1.50	.140
	Reexperiencing	0.370	2.28	.026
	Age	0.231	1.94	.057
	Sex[F]	0.314	2.64	.011
	Total thalamus	0.088	0.71	.478
Motor	Anxious arousal	–0.541	–3.90	**.0002**
	Avoidance	0.206	1.30	.199
	Dysphoric arousal	–0.160	–0.82	.417
	Emotional numbing	0.201	1.22	.227
	Reexperiencing	0.442	2.73	**.008**
	Age	0.146	1.22	.225
	Sex[F]	0.358	3.00	**.004**
	Total thalamus	0.196	1.59	.117

*Note*: All models had a significant intercept with *p* <.0001.

### 3.3 Examination of potentially confounding variables

When covarying for BDI score, both reexperiencing and anxious arousal scores remained significant predictors in all five regressions. Moreover, BDI score had a nonsignificant effect in all the regressions. Similarly, covarying for LEC or CTQ scores did not impact the significance of reexperiencing and anxious arousal scores in any model, and neither LEC nor CTQ score was a significant effect in any of the five models.

## 4 DISCUSSION

This study identified relationships of reexperiencing and anxious arousal PTSD symptom dimensions with volumes of functional groupings of thalamic nuclei. The results do not support our hypothesis that reexperiencing symptoms would track primarily with volumes of thalamic nuclei involved in sensory processing. Instead, we found significant positive correlations of reexperiencing with volumes of ventral thalamic nuclei involved in motor functions, namely ventral anterior, ventral lateral, and ventromedial nuclei. In addition, we found that anxious arousal symptoms were negatively correlated with volumes of all five thalamic nuclei groupings examined, indicating a broad relevance of thalamus subregions to arousal alterations in PTSD. These relationships were seen with all PTSD symptom dimensions entered as simultaneous predictors of thalamus volumes and were not attributable to lifetime trauma load or depression severity. None of the other PTSD symptom dimensions—dysphoric arousal, avoidance, emotional numbing—were significantly associated with any of the thalamus grouping volumes.

Counter to prediction, we did not find a relationship of reexperiencing symptoms with volumes of sensory thalamic nuclei. The volumes of the specific sensory and associative‐sensory thalamic nuclei groupings were not significantly associated with reexperiencing symptoms after Bonferroni correction. Specific sensory thalamic nuclei are first‐order sensory relay nuclei, including the geniculate nuclei and ventral posterolateral nuclei, which receive inputs from ascending pathways and subcortical brain regions, and then transmit primary sensory information to cortical areas (Noback et al., 2005). Associative‐sensory nuclei, including the pulvinar and lateral posterior nucleus, are involved in transmission of sensory information across dorsal attention and visual cortical networks, and contribute to multimodal sensory integration and attentional filtering of salient stimuli (Schmahmann, 2003; Theyel et al., 2010). Despite our study's parcellation of these two functional groupings of sensory thalamic nuclei, these groupings still represent some functional heterogeneity across and within their component individual nuclei. For instance, the specific sensory nuclei each specialize in a distinct sensory modality, such as the lateral geniculate's role in the primary visual system. Similarly, although it is established that most of the pulvinar nucleus is involved in vision, there is also evidence that the inferior pulvinar and certain parts of lateral pulvinar nuclei are more densely connected with striate and extrastriate cortex, whereas the medial pulvinar and other areas of the lateral pulvinar are associated with higher‐order cortices (Grieve et al., 2000). Thus, it is possible that a more fine‐grained subdivision of the sensory thalamus would yield different findings regarding relationships with reexperiencing and other PTSD symptoms. This could be examined in future studies that have sufficient statistical power to parse a larger number of nuclei.

Reexperiencing symptoms showed a strong and significant positive association with volumes of the motor thalamic nuclei (Table 3). To our knowledge, this is the first report implicating these nuclei in PTSD phenomenology. These nuclei on the ventral side of the thalamus facilitate multiple simple and complex motor functions (Huys et al., 2016; Takahashi et al., 2021), and they are treatment targets for deep brain stimulation therapies of movement disorders (Casagrande et al., 2019). Interestingly, one of the functions of the motor thalamic group is to support oculomotion (eye movements), which could be relevant to our finding that volumes of motor nuclei correlated with reexperiencing symptoms of PTSD. Indeed, eye movements are known to modulate the retrieval of autobiographical memories, and eye movement desensitization and reprocessing (EMDR) therapy for PTSD leverages the influence of eye movements on the retrieval and reprocessing of traumatic memories (Lee & Cuijpers, 2013). During EMDR, patients recall their traumatic experience while making eye movements, leading to diminished vividness and emotionality of the memories (Andrade et al., 1997; Barrowcliff et al., 2004; Cotter et al., 2017). Although early functional imaging studies of brain changes during EMDR treatment converged on the involvement of frontal regions (Lansing et al., 2005; Oh & Choi, 2007; Pagani et al., 2012), more recent evidence also implicates modification of thalamus activity in EMDR‐mediated PTSD symptom improvement (Rousseau et al., 2019). We suggest that our finding of motor thalamic nuclei volumes correlating with reexperiencing symptoms motivates examination of whether these nuclei are part of the extended brain network that mediates interactions between eye movements and trauma memory retrieval in PTSD.

Anxious arousal symptoms were highly negatively correlated with volumes of all the thalamic nuclei groupings (Table 3), including those implicated in sensory processing, general arousal, motor functions, and memory and cognition. This seems consistent with preclinical and clinical evidence that physiological arousal has a broad role in modulating attentional, sensory, and cognitive functions (O'Donnell et al., 2004). Negative correlations between thalamus imaging measures and hyperarousal symptoms also have been found using functional imaging. For instance, during script‐driven imagery, lower thalamus activation is seen among PTSD patients who report a larger subjective hyperarousal response to traumatic scripts (Lanius et al., 2006). Moreover, a meta‐analysis of seven functional imaging studies of Pavlovian fear conditioning concluded that PTSD patients compared with trauma‐exposed controls fail to activate the thalamus during all phases of fear and extinction learning (Suarez‐Jimenez et al., 2019). Consistent with known involvement of the thalamus in arousal and visual attention (Greene et al., 2020; Noback et al., 2005), deficient functional activation of the thalamus may explain patients’ difficulty using external cues to regulate their arousal levels, contributing to hypervigilance and exaggerated arousal symptoms (Suarez‐Jimenez et al., 2019). The associations found in the present study between anxious arousal and volumes of all thalamus groupings may be consistent with a role of arousal systems as important for multiple behavioral features of PTSD (Ressler & Nemeroff, 2000).

Strengths of this study include the use of semistructured interviews administered by doctoral‐level psychologists, and the inclusion of a range of PTSD spectrum severity extending to subthreshold symptom presentations. In addition, we can be confident that our findings do not reflect medication effects, since all but one of the participants were nonmedicated. However, our findings are cross‐sectional and correlational, and they cannot discern whether differences in thalamic nuclei volumes may lead to particular symptom presentations, or vice versa, or whether the identified relationships are driven by a third variable. Another limitation is that our sample size precludes separating the participants into subgroups based on severity; it is possible for relationships between psychiatric symptoms and biological correlates to be nonlinear, with one or more “tipping points” that mark a transition to more severe psychopathology (Cuthbert & Insel, 2013). However, there has not been sufficient research to determine the “location” of such points, including whether any of them aligns with the categorical diagnostic threshold for PTSD. Accordingly, this will be a question to elucidate with larger samples. Finally, the data were collected under DSM‐IV rather than DSM‐5 criteria due to the timing of the study. This is a limitation for generalization to the current diagnostic system, although we used of a 5‐factor model of PTSD symptoms rather than the 3‐factor DSM‐IV model. Overall, these findings could be strengthened and extended by replicating them in a dataset with a larger sample, which also would permit a more fine‐grained parcellation of thalamic nuclei due to higher statistical power.

To summarize, in this sample of posttraumatic stress spectrum adults, we found reexperiencing symptoms to be significantly positively correlated with volumes of ventral thalamic nuclei that mediate motor functions. One of these motor functions, oculomotion, may be implicated in autobiographical and trauma memory recall based on prior research. Anxious arousal symptoms were correlated with volumes of all thalamic groupings, consistent with extant knowledge that the thalamus is involved in a broad range of behavioral components of PTSD, which all can be modulated by physiological arousal. Overall, these findings encourage further examination of the functionally and anatomically diverse thalamus nuclei with the complex presentation of PTSD.

## CONFLICT OF INTEREST

None of the authors have a financial conflict of interest to report.

### PEER REVIEW

The peer review history for this article is available at https://publons.com/publon/10.1002/brb3.2639

## Data Availability

Data available on request from the authors.
